# *Fosb* Induction in Nucleus Accumbens by Cocaine Is Regulated by E2F3a

**DOI:** 10.1523/ENEURO.0325-18.2019

**Published:** 2019-04-01

**Authors:** Hannah M. Cates, Casey K. Lardner, Rosemary C. Bagot, Rachael L. Neve, Eric J. Nestler

**Affiliations:** 1 Nash Family Department of Neuroscience and Friedman Brain Institute, Icahn School of Medicine at Mount Sinai, New York, New York 10029; 2 Gene Delivery Technology Core, Massachusetts General Hospital, Cambridge, Massachusetts 02139

**Keywords:** addiction, cocaine, E2F3a, ∆FosB, nucleus accumbens, transcription

## Abstract

The transcription factor ΔFosB has been proposed as a molecular switch for the transition from casual, volitional drug use into a chronically addicted state, but the upstream regulatory mechanisms governing ΔFosB expression are incompletely understood. In this study, we find a novel regulatory role for the transcription factor E2F3, recently implicated in transcriptional regulation by cocaine, in controlling ΔFosB induction in the mouse nucleus accumbens (NAc) following cocaine administration. We find that an E2F consensus sequence 500 bp upstream of the *Fosb* transcription start site is enriched for E2F3 specifically over other E2F isoforms. We further conclude that ΔFosB expression is regulated specifically by E2F3a, not E2F3b, that *E2f3a* expression is specific to D1 receptor-expressing medium spiny neurons, and that E2F3a overexpression in NAc recapitulates the induction of *Fosb* and *ΔFosb* mRNA expression observed after chronic cocaine exposure. E2F3a knockdown in NAc does not abolish *ΔFosb* induction by cocaine, a result consistent with previously published data showing that singular knockdown of upstream regulators of ΔFosB is insufficient to block cocaine-induced expression. Finally, to elucidate potential combinatorial epigenetic mechanisms involved in E2F3a’s regulation of *Fosb*, we explore H3K4me3 enrichment at the *Fosb* promoter and find that it is not enhanced by E2F3a overexpression, suggesting that it may instead be a pre-existing permissive mark allowing for E2F3a to interact with *Fosb*. Together, these findings support a role for E2F3a as a novel, upstream regulator of the addiction-mediating transcription factor ΔFosB in NAc.

## Significance Statement

A hallmark question in the neuroscience of addiction is how repeated drug use is translated into long-lasting changes in neural regulation. Long-lasting changes in behavior are mediated in part by intracellular transcriptional mechanisms which affect gene expression and neural circuitry. Therefore, pinpointing signaling pathways downstream of abused drugs is a high priority for understanding the addiction process. To this end, we identify and characterize E2F3a as a novel regulator of ΔFosB, a transcription factor widely studied as a molecular switch for the transition from casual drug use to addiction.

## Introduction

Drug addiction is a partly heritable disorder, with ∼50% of risk explained by genetic sequence variations (Meyre and Mayhew, 2017). Thus, many preclinical and clinical studies have focused on epigenetic effects and gene expression differences induced by chronic exposure to drugs of abuse. The transcription factor ΔFosB, an alternative splice variant encoded by the *Fosb* gene, accumulates in nucleus accumbens (NAc) after repeated exposure to virtually all abused substances and thus has been proposed as a molecular switch for the transition from casual drug use into the chronically addicted state by reorganizing gene expression (Nestler, 2015). This phenomenon positions ΔFosB and its encoding gene *Fosb* as unique therapeutic targets.

Numerous studies have demonstrated the necessity and sufficiency of ΔFosB for molecular and behavioral responses to cocaine and other stimulants, and have focused on downstream genes controlled by ΔFosB induction in stimulant addiction (Hiroi et al., 1997; Carlezon et al., 1998; Kelz et al., 1999; McClung and Nestler, 2003; Renthal et al., 2008, 2009; Maze et al., 2010; Grueter et al., 2013; Heller et al., 2014). However, little is known about how ΔFosB induction itself is regulated downstream of drug action. Clarifying mechanisms of ΔFosB induction in the context of stimulant addiction is critical for further understanding how this transcription factor contributes to the pathogenesis of addiction. To this end, this study uses cocaine exposure to investigate a putative transcriptional mediator of ΔFosB induction in NAc.

A prior study that used ChIP-seq and RNA-seq in parallel to investigate unbiased, genome-wide transcriptional mechanisms underlying persistent changes in gene expression in mouse NAc after repeated cocaine administration identified the E2F family of transcription factors as among the most highly implicated upstream regulator of both gene expression and alternative splicing (Feng et al., 2014). A more recent study showed that E2F3a, one of two E2F3 isoforms, mediates a substantial portion of changes in gene expression and alternative splicing in NAc after chronic cocaine exposure as well as promotes behavioral actions of the drug (Cates et al., 2018b). Given these findings, we hypothesized that E2F3a might also regulate ΔFosB expression and splicing.

**Figure 1.**
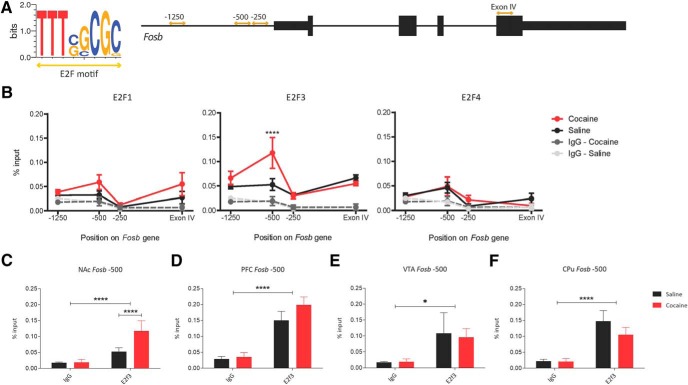
E2F3 binding at the *Fosb* gene promoter is regulated in NAc by cocaine. ***A***, E2F consensus sequence and a schematic diagram of *Fosb*. Primer sets designed against putative E2F binding sites noted with golden double arrowheads. ***B***, E2F3 binding, not E2F1 or E2F4, was increased following cocaine administration 500 bp upstream of the *Fosb* TSS (interaction of drug and gene position was observed by two-way ANOVA: *F*_(9,60)_ = 13.35, *p* < 0.0001; Cocaine vs saline at −500 bp via *post hoc* analysis: *t*_(8)_ = 9.904, ****p < 0.0001). All data points have error bars representing ± SEM. ***C***–***F***, Significant binding of E2F3 over an IgG control was observed and significantly increased by cocaine in (**C**) NAc (*F*_(9,60)_ = 13.35, ****p < 0.0001, *t*_(8)_ = 9.904, ****p < 0.0001). Significant binding of E2F3 was observed but was not affected by cocaine administration in (***D***) PFC, binding over an IgG control (*F*_(1,16)_ *=* 48.44, ****p < 0.0001), no main effect of drug observed (*F*_(1,16)_ = 1.789, *p* = 0.1998); (***E***) VTA, binding over an IgG control (*F*_(1,10)_ = 7.024, *p = 0.0243), no main effect of drug observed (*F*_(1,10)_ = 0.029, *p* = 0.869); or (***F***) CPu, binding over an IgG control (*F*_(1,16)_ = 25.113, ****p < 0.0001), no main effect of drug observed (*F*_(1,16)_ = 1.12, *p* = 0.305). *N* = 4–5, with three mice pooled per sample for all qChIP assays.

ΔFosB arises via an alternative splicing event of *Fosb* mRNA in which Exon IV is truncated (Alibhai et al., 2007; Fig. 1*A*). Because two degradation domains are cleaved in the truncation of Exon IV, ΔFosB is unusually stable and accumulates on repeated *Fosb* induction (Nye and Nestler, 1996; Carle et al., 2007). Furthermore, phosphorylation of Ser27 in ΔFosB following cocaine exposure leads to further enhanced stability (Ulery-Reynolds et al., 2009). Although some factors leading to ΔFosB’s induction and accumulation have been elucidated—polypyrimidine tract binding protein 1 (PTB; encoded by *Ptbp1*), cAMP response element binding protein (CREB), serum response factor (SRF), and early growth response factor 3 (EGR3; Marinescu et al., 2007; Vialou et al., 2012; Chandra et al., 2015)—many of the upstream regulators and mechanisms governing ΔFosB expression following drug exposure remain to be clarified. Here, we examine the influence of E2F3a on ΔFosB expression in four brain regions related to processing the molecular and behavioral effects of cocaine: NAc, caudate–putamen (CPu), ventral tegmental area (VTA), and prefrontal cortex (PFC). Our goal is to gain insight into the regulation of this key upstream regulator of cocaine-mediated neural and behavioral changes.

E2F transcription factors are involved in chromatin modification, gene regulation, and RNA processing via direct interaction with and recruitment of histone modifying enzymes and splicing machinery, as well as binding directly to splice sites and influencing the expression of splicing factors (Lees et al., 1993; Ahlander and Bosco, 2009). E2F3 has two alternative splice forms, E2F3a and E2F3b, which have been shown to be activating or repressive under different conditions (Leone et al., 2000). Like all E2F family members, E2F3a and E2F3b share a canonical consensus sequence “TTTCGCGC” (Reichel et al., 1987; Hume et al., 2015). Here, we demonstrate that E2F3a is a key upstream regulator of *Fosb*, and induces expression of the canonical addiction-mediating transcription factor, ΔFosB. Specifically, we show that E2F3 binds at an E2F consensus sequence 500 bp upstream of the *Fosb* transcription start site (TSS) and that E2F3a, but not E2F3b, expression is increased in dopamine receptor type 1 (D1)-expressing medium spiny neurons (MSNs) following cocaine exposure. Further, overexpression of E2F3a, but not E2F3b, recapitulates induction of ΔFosB as seen with repeated cocaine administration. These data establish a novel role for E2F3a in the brain in regulating the molecular response to cocaine, specifically in regulating expression of the transcription factor ΔFosB in NAc.

## Materials and Methods

### Animals and treatments

C57BL/6J male mice (The Jackson Laboratory), 7–8 weeks old and weighing 25–30 g, were habituated to the animal facility 1 week before use and maintained at 22–25°C on a 12 h light/dark cycle. All animals had access to food and water *ad libitum*. All animal procedures were performed in accordance with guidelines of the Institutional Animal Care and Use Committee at the Icahn School of Medicine at Mount Sinai.

For quantitative chromatin immunoprecipitation (qChIP) assays and gene expression measures following fluorescence activated cell sorting (FACS), mice were injected intraperitoneally with saline or cocaine (20 mg/kg) once daily for 7 d in their home cage. Mice were then euthanized 24 h following the final cocaine injection.

For assaying the effects of viral-mediated E2F3a knockdown, mice were injected intraperitoneally with saline once daily for 4 d beginning 2 d post-viral infusion. On the fifth day, animals were either injected with a final dose of saline or a single, acute dose of cocaine (20 mg/kg) and euthanized 1 h later.

### Viral-mediated gene transfer

Mice were anesthetized with a mixture of ketamine (100 mg/kg) and xylazine (10 mg/kg) and prepared for stereotactic surgery. Thirty-three gauge syringe needles (Hamilton) were used to bilaterally infuse 0.5 µl virus in NAc at a rate of 0.1 µl/min at 1.6 mm anterior, 1.5 mm lateral, and 4.4 mm ventral from bregma at a 10° angle, or in PFC at 1.8 mm anterior, 0.75 mm lateral, and 2.7 mm ventral from bregma at a 15° angle. We used bicistronic p1005 HSV vectors expressing GFP alone or GFP plus E2f3a or E2f3b for overexpression experiments, or HSV vectors that expressed GFP as well as microRNAs targeted against LacZ or *E2f3a* for knockdown experiments. In these systems, GFP expression is driven by a cytomegalovirus promoter, whereas the select gene of interest is driven by the IE4/5 promoter (Maze et al., 2010). Viral expression was confirmed during tissue collection using fluorescence microscopy (Leica) to visualize GFP and confirm targeting of NAc or PFC. The effectiveness of the overexpression and knock-out vectors in NAc has been published (Cates et al., 2018b) and replicated here (data not shown).

### Tissue collection

Tissue was collected at the appropriate time point following pharmacological or viral manipulation via cervical dislocation followed by dissection of NAc with a 14-gauge blunt needle for cocaine studies, or a 15-gauge needle for viral studies to ensure only virally infected tissue was dissected. PFC was dissected with a single central 12-gauge punch for cocaine studies, or a 14-gauge punch for viral studies. Tissue was immediately frozen on dry ice. Single animal samples were used in all RNA-qPCR experiments. For qChIP experiments, animals were pooled as described below.

### Quantitative chromatin immunoprecipitation

qChIP was performed on NAc or PFC punches, pooled from three mice, 72 h after viral infection, or from two mice, 24 h after the last drug treatment. qChIP methodology was modified from Ferguson et al. (2015). Chromatin was prepared by fixation in 940 µl 1% formaldehyde for 12 min followed by addition of 64 µl 2 m glycine to stop the fixing reaction for 5 min. Punches were then washed 5 times in cold PBS, and passed through a 21-gauge syringe 10 times to homogenize the tissue. The homogenized tissue was centrifuged at 1250 rcf, and then resuspended and incubated in a 0.5% NP-40 cellular lysis buffer for 15 min, centrifuged at 2700 rcf and resuspended in a 1% SDS nuclear lysis buffer for 10 min. The samples were then sheared using a Diagenode Bioruptor XL at high sonication intensity for 30 s on/30 s off for 10 min twice (to prevent overheating). Fragment size and chromatin DNA concentration was verified with an Agilent 2100 Bioanalyzer at 150–300 bp. Sheared chromatin was incubated overnight in a 0.1% SDS solution with the following antibodies previously bound to magnetic beads following the Dynabead protocol (Dynabeads M-280, Life Technologies): antibody to E2F3 [Santa Cruz Biotechnology, E2F-3 (C-18) × (sc-878X)], antibody to Rabbit-IgG [Santa Cruz Biotechnology, normal rabbit IgG × (sc-2027X)], antibody to H3K4me3 [ActiveMotif, Histone H3K4me3 antibody (pAb); 39915]. 1 µg of DNA was used for E2F3 and IgG IPs, 250 ng for H3K4me3. After incubation, beads were washed four times: (1) low salt (150 mm NaCl), (2) high salt (500 mm NaCl), (3) 150 mm LiCl, and (4) TE + 50 mm NaCl. Chromatin was eluted off the beads using a 100 mm sodium bicarbonate/1% SDS solution at 65°C for 30 min. Then reverse crosslinking took place overnight at 65°C, and DNA purification was performed via Qiagen Spin Columns. Binding to the *Fosb* gene was determined by qRT-PCR using primers detailed in Table 1. The E2F consensus sequence was identified using MotifMap (Daily et al., 2011). The binding sites investigated were determined using MatInspector, which predicts functional transcription factor binding sites using a position weight matrix approach to identify known binding motifs and compute a likelihood of actual functional binding determined by the surrounding base pair context (Cartharius et al., 2005).

**Table 1.** ChIP-qPCR Primers along the *Fosb* gene

**Location on *Fosb***	**Forward**	**Reverse**
*−1250*	ATGGGACTCAGGTTGTCAGG	AGCCAGGGCTACACAGAGAA
*−500*	GAGTTGCACCTTCTCCAACC	GGCCCAGTGTTTGTTTGGTA
*−250*	ATGGCTAATTGCGTCACAGG	ACCTCCCAAACTCTCCCTTC
*Exon IV*	CAACCTGACGGCTTCTCTCT	CGGGTTTGTTTGTTTTGTTTG

These qChIP primers were used to amplify three putative E2F consensus sequences in *Fosb:* 500 and 250 bp upstream of the TSS, and a site within Exon IV. A sequence 1250 bp upstream of the TSS was used as a negative control.

### FACS isolation of MSNs

Male D1-Tomato and D2-GFP were used to tag D1-receptor expressing and dopamine 2 (D2)-receptor expressing MSNs, respectively, with tdTomato or GFP fluorophores. Whole, tagged neurons were isolated and collected as previously described (Engmann et al., 2017). Briefly, NAc punches were collected in ice-cold digestion buffer, digested in papain solution, and titrated to allow for cells to gently disperse. Intact, dissociated cells were selected by passing the samples over an ovomucoid gradient. Sorting was performed on a BD FACS Aria II and collected into Trizol LS-reagent (Ambion, 10296-028) and RNA was extracted using the Direct-zol RNA miniprep kit with DNase treatment (Zymo, R2050), following the manufacturer’s recommendations. RNA quality was assessed with an Agilent RNA 6000 PICO kit (Agilent Technologies, 5067-1513).

### RNA isolation and qPCR

RNA isolation, qPCR and data analysis were performed as previously described (Maze, et al., 2010). Briefly, RNA was isolated with TriZol reagent (Invitrogen) and was further purified with RNAeasy microkits from Qiagen. Samples were analyzed on a NanoDrop 2000 to assess RNA purity (260:280 ratio between 1.80 and 2 and 260/230 > 1). Three hundred nanograms of RNA were converted to cDNA using iScript (Bio-Rad) according to the manufacturer’s protocol, and qPCR was conducted using SYBR green (Quanta) with an Applied Biosystems 7900HT RT PCR system using the following cycle parameters: 2 min at 95°C; 40 cycles of 95°C for 15 s, 59°C for 30 s, 72°C for 33 s; and graded heating to 95°C to generate dissociation curves for confirmation of single PCR products. Data were analyzed by comparing *C_t_* values of the treatment condition to the control condition with the ΔΔ*C_t_* method (Tsankova et al., 2006). Primer sequences are listed in Table 2.

**Table 2.** RT-qPCR Primers for *Fosb* transcripts and *Gapdh* for probing mRNA expression

**Transcript**	**Forward**	**Reverse**
*Fosb*	GTGAGAGATTTGCCAGGGTC	AGAGAGAAGCCGTCAGGTTG
*ΔFosb*	AGGCAGAGCTGGAGTCGGAGAT	GCCGAGGACTTGAACTTCACTCG
*Gapdh*	AGGTCGGTGTGAACGGATTTG	TGTAGACCATGTAGTTGAGGTCA

These qPCR primers were used to amplify and assess expression of *Fosb* and *ΔFosb* mRNA. *Gapdh* was used as a housekeeping gene in the ΔΔ*C_t_* method.

### Statistics

All analyses were performed using Prism software (GraphPad). Student’s *t* tests were used for any pairwise comparisons, and two-way ANOVAs were used for all multiple comparisons, followed by Bonferroni *post hoc* tests where appropriate. A type I error significance level of 0.05 was used to determine statistical significance for all calculations.

## Results

### Regulation of *Fosb* by E2F3 after cocaine administration in NAc is both isoform- and region-specific

We identified, through a detailed investigation of the *Fosb* gene using MatInspector, three putative E2F binding sites (Daily et al., 2011; Cartharius et al., 2005). Two are in the promoter ∼250 and 500 bp upstream of the TSS. A third is in Exon IV near the alternative splice site where the truncation of full-length *Fosb* mRNA occurs and leads to expression of *ΔFosb* mRNA (Fig. 1*A*). To test whether *Fosb* and thus ΔFosB expression is regulated by E2F3 specifically, and not another E2F isoform, we performed qChIP for E2F1, E2F3, and E2F4 in NAc under both saline and repeated cocaine conditions (once daily intraperitoneal injections for 7 d). E2F2, E2F5, E2F6, E2F7, and E2F8 are sparsely expressed in the adult mouse brain (Lein et al., 2007) and were not investigated in this study. We designed primers to amplify the three identified binding sites of interest, as well as a negative control region where no binding was expected at 1250 bp upstream of the TSS. An interaction of cocaine treatment and gene position was observed for E2F3 only (*F*_(9,60)_ = 13.35, *p* < 0.0001; *N* = 4–5, 3 mice/sample for all qChIP assays; row a, Table 3), with a specific increase in binding following cocaine administration at the site 500 bp upstream of the TSS (*t*_(8)_ = 9.904, *p* < 0.0001; Fig. 1*B*,*C*; row a, Table 3). Note that no available antibody can distinguish between E2F3a and E2F3b.

**Table 3.** Statistical table

	**Figure**	**Data structure**	**Type of test**	**Power**
a	Fig. 1*B*,*C*	Normal distribution	4 × 4 ANOVA	0.0621
b	Fig. 1*D*	Normal distribution	2 ×2 ANOVA	0.0595
c	Fig. 1*E*	Normal distribution	2 ×2 ANOVA	0.0595
d	Fig. 1*F*	Normal distribution	2 ×2 ANOVA	0.0595
e	Fig. 2*A*	Normal distribution	2 ×2 ANOVA	0.0558
f	Fig. 2*B*	Normal distribution	2 ×2 ANOVA	0.0558
g	Fig. 2*D*	Normal distribution	2 ×3 ANOVA	0.0692
h	*ΔFosb/Fosb* mRNA ratio	Normal distribution	Student’s *t* test	0.0661
i	Fig. 2*E*	Normal distribution	2 ×2 ANOVA	0.0607
j	Fig. 2*F*	Normal distribution	2 ×4 ANOVA	0.0651
k	Fig. 3*A*	Normal distribution	2 ×2 ANOVA	0.0607
l	Fig. 3*B*	Normal distribution	2 ×2 ANOVA	0.0607

Next, to determine whether regulation of *Fosb* by E2F3 at this −500 bp site is specific to NAc, we repeated qChIP for E2F3 in three additional regions of the reward circuitry: PFC, VTA, and CPu. In PFC, significant binding over an IgG control was observed (*F*_(1,16)_ *=* 48.44, *p* < 0.0001; *N* = 5; row b, Table 3), but this effect was not significantly different between saline and cocaine conditions (*t*_(8)_ = 1.69, *p* = 0.2208; Fig. 1*D*; row b, Table 3). Modest binding was observed in VTA, but again this was not significantly different between saline and cocaine (*F*_(1,10)_ = 7.024, *p* = 0.0243; *t*_(5)_ = 0.275, *p* = 0.9999; *N* = 3–4; Fig. 1*E*; row c, Table 3). E2F3 binding in CPu was also significantly increased over an IgG control (*F*_(1,16)_ = 25.113, *p* < 0.0001; *N* = 5; row d, Table 3), with no significant effect of cocaine (*t*_(8)_ *=* 1.451, *p* = 0.3324; Fig. 1*F*; row d, Table 3). Together, these data indicate that regulation of E2F3 binding to *Fosb* after repeated cocaine is specific to NAc.

### Expression of E2F3a, not E2F3b, is increased by cocaine in NAc D1 MSNs

**Figure 2.**
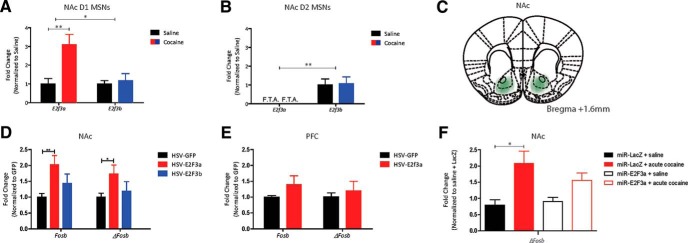
E2F3a is expressed in NAc D1 MSNs and overexpression of E2F3a in NAc regulates expression of *Fosb* isoforms in NAc. ***A***, Expression of *E2f3a* and *E2f3b* in FACS-isolated D1 MSNs. Main effects of both drug treatment (*F*_(1,9)_ *=* 9.624, *p* = 0.0127) and transcript variant (*F*_(1,9)_ *=* 6.845,*p = 0.028) were observed. A significant increase in *E2f3a* expression was confirmed via *post hoc* analysis (*E2f3a: t*_(5)_ = 4.186, **p = 0.0047; *E2f3b: t*_(4)_ = 0.3328, *p* = 0.9999). *N* = 3–4. ***B***, Expression of *E2f3a* and *E2f3b* in FACS-isolated D2 MSNs. A main effect of transcript was observed (*F*_(1,9)_ *=* 15.489, **p = 0.0034) but no effect of drug treatment (*F*_(1,9)_ *=* 0.0171, *p* = 0.8987). *N* = 3–4. ***C***, NAc cross section indicating site of injection for viral-mediated gene transfer. ***D***, qPCR from mouse NAc injected with HSV-GFP, HSV-E2F3a, or HSV-E2F3b. Normalized to GFP (*Gapdh* used as housekeeping gene in ΔΔ*C_t_* method). A main effect of virus was observed (*F*_(2,29)_ = 9.332, *p* = 0.0007). E2F3a, but not E2F3b, increased both *Fosb* and *ΔFosb* mRNA expression by *post hoc* analysis (E2F3a vs GFP–*Fosb*: *t*_(10)_ = 3.138, **p = 0.0117; E2F3a vs GFP–*ΔFosb*: *t*_(9)_ = 2.868, *p = 0.0229; E2F3b vs GFP–*Fosb*: *t*_(10)_ = 1.336, *p* = 0.5757; E2F3b vs GFP–*ΔFosb*: *t*_(10)_ = 0.575, *p* = 0.9999; *N* = 8). ***E***, qPCR from mouse PFC injected with HSV-GFP or HSV-E2F3a. Normalized to GFP. No main effect of virus was observed (*F*_(1,19)_ = 1.637, *p* = 0.2162; *N* = 6). ***F***, qPCR from mouse NAc injected with HSV-miR-LacZ or HSV-miR-E2F3a and treated with either saline or acute cocaine (1 injection of 20 mg/kg). Normalized to miR-LacZ + saline. A main effect of drug treatment was observed (*F*_(3,20)_ = 3.297, *p* = 0.0415) and *post hoc* analysis confirmed that acute cocaine increased *ΔFosb* expression and viral knockdown of E2F3a did not significantly blunt this effect (miR-LacZ + saline vs miR-LacZ + cocaine: *t*_(9)_ = 2.492, *p = 0.0508, miR-LacZ + saline vs miR-E2f3a + cocaine: *t*_(9)_ = 1.223, *p* = 0.9999; miR-LacZ + cocaine vs miR-E2f3a + cocaine: *t*_(10)_ *=* 1.331, *p* = 0.9999; *N* = 5–7).

To explore the cell-type-specific expression of *E2f3*’s two isoforms, E2F3a and E2F3b, we performed FACS on tagged D1 MSNs or D2 MSNs and analyzed the mRNA expression of each transcript. In isolated D1 MSNs, both *E2f3a* and *E2f3b* are expressed at baseline levels, but only *E2f3a* expression is increased by repeated cocaine exposure. Main effects of both drug treatment (*F*_(1,9)_ *=* 9.624, *p* = 0.0127; row e, Table 3) and transcript variant (*F*_(1,9)_ *=* 6.845, *p* = 0.028; *N* = 3–4; row e, Table 3) were observed and a significant increase in *E2f3a* expression only was confirmed via *post hoc* analysis (*E2f3a: t*_(5)_ = 4.186, *p* = 0.0047; *E2f3b: t*_(4)_ = 0.3328, *p* = 0.9999*;* Fig. 2*A*; row e, Table 3). In D2 MSNs, *E2f3b* is expressed minimally but no *E2f3a* expression was detected under control or cocaine conditions (*F*_(1,9)_ *=* 15.489, *p* = 0.0034; *N* = 3–4; Fig. 2*B*; row f, Table 3). Importantly, these data indicate that *E2f3a* is induced by cocaine selectively in the same neuronal population in which cocaine induction of ΔFosB occurs. Thus, Lobo et al. (2013) found that ΔFosB is induced by cocaine in D1 but not D2 NAc MSNs, and Grueter et al. (2013) demonstrated that selective overexpression of ΔFosB in D1 but not D2 NAc MSNs enhances behavioral responses to cocaine.

### Overexpression of E2F3a recapitulates cocaine-induced *Fosb* and *ΔFosb* mRNA expression

To probe which E2F3 isoform regulates ΔFosB expression, we performed stereotactic surgery to infuse HSV-GFP, -E2F3a, or -E2F3b into NAc. A main effect of virus was observed (*F*_(2,29)_ = 9.332, *p* = 0.0007; *N* = 6; row g, Table 3), and a significant E2F3a-induced increase in both *Fosb* and *ΔFosb* mRNA expression was confirmed via *post hoc* analysis (*Fosb*: *t*_(10)_ = 3.138, *p* = 0.0078; *ΔFosb*: *t*_(9)_ = 2.868, *p* = 0.0185; Fig. 2*D*; row g, Table 3). The ratio of *Fosb*–*ΔFosb* mRNA was not affected by E2F3a overexpression (*t*_(9)_ = 0.3142, *p* = 0.7605; row h, Table 3), consistent with the lack of binding of E2F3 to the *Fosb* splice site in Exon IV. E2F3b overexpression did not affect mRNA expression of either *Fosb* isoform (*Fosb*: *t*_(10)_ = 1.336, *p* = 0.5757; *ΔFosb*: *t*_(10)_ = 0.575, *p* = 0.9999; Fig. 2*D*; row g, Table 3).

Because significant binding of E2F3 was observed in PFC (Fig. 1*E*), but was unaffected by cocaine, we used this region as a control for the effect of E2F3a overexpression in NAc. E2F3a overexpression in PFC did not affect expression of either *Fosb* or *ΔFosb* mRNA (*F*_(1,19)_ = 1.637, *p* = 0.2162; *N* = 6; Fig. 2*E*; row i, Table 3).

To further explore the necessity of E3F3a binding to *Fosb* to induce *ΔFosb* expression, we knocked down expression of E2F3a with a miRNA and assessed *ΔFosb* mRNA levels following an acute dose of experimenter-administered cocaine. We found a main effect of treatment (*F*_(3,20)_ = 3.297, *p* = 0.0415; *N* = 5–7; row j, Table 3), and confirmed via *post hoc* analysis that an acute dose of cocaine induces a near-significant increase in *ΔFosb* mRNA expression (miR-LacZ + saline vs miR-LacZ + cocaine: *t*_(9)_ = 2.492, *p* = 0.0508; Fig. 2*F*; row j, Table 3). On E2F3a knockdown, cocaine no longer induced *ΔFosb* (miR-LacZ + saline vs miR-E2f3a + cocaine: *t*_(9)_ = 1.223, *p* = 0.6855; row j, Table 3), but the difference between E2F3a knockdown and control conditions did not achieve statistical significance (miR-LacZ + cocaine vs miR-E2f3a + cocaine: *t*_(10)_ = 1.3311, *p* = 0.9999; Fig. 2*F*; row j, Table 3). Together, these findings indicate that E2F3a binding to the *Fosb* promoter is sufficient for induction of *ΔFosb* expression but is not solely required. This is consistent with published data on two other transcription factors that regulate ΔFosB: SRF and CREB. Ablation of both of these upstream regulators simultaneously blocks the cocaine-induced increase in *ΔFosb*, but knockdown of either molecule alone is insufficient (Vialou et al., 2012).

### Overexpression of E2F3a increases binding of E2F3 at the *Fosb* promoter but does not affect H3K4me3 enrichment

**Figure 3.**
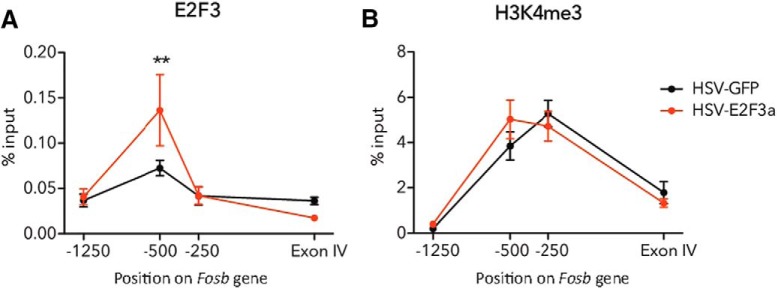
Viral-mediated E2F3a overexpression leads to increased E2F3 binding at *Fosb* promoter in NAc. ***A***, E2F3 binding was increased at 500 bp upstream of the *Fosb* TSS after E2F3a overexpression (interaction of virus and gene position by two-way ANOVA: *F*_(3,35)_ = 3.619, *p* = 0.0224; E2F3a vs GFP at −500 bp via *post hoc* analysis: *t*_(7)_ = 3.285, **p = 0.0093). ***B***, H3K4me3 enrichment was not affected by E2F3a overexpression (no main effect of virus observed: *F*_(1,40)_ = 0.057, *p* = 0.8126). *N* = 5–6, with three mice pooled per sample for all qChIP assays.

To determine whether this increase in *Fosb* expression following E2F3a overexpression is because of direct binding at the *Fosb* gene promoter in NAc, qChIP was performed following overexpression of E2F3a. An interaction of virus and gene position was observed following overexpression of E2F3a (*F*_(3,35)_ = 3.619, *p* = 0.0224; *N* = 5–6, 3 mice/sample for all qChIP assays; row k, Table 3). E2F3a overexpression elicited a specific increase in E2F3 binding 500 bp upstream of the TSS, the same site at which binding was observed following cocaine exposure, but not at the two other putative binding sites as confirmed by *post hoc* analysis (*t*_(7)_ = 3.285, *p* = 0.0093; Fig. 3*A*; row k, Table 3). This result, along with our initial cocaine qChIP results, confirms that the putative E2F binding site 500 bp upstream of the TSS is a functional E2F response element, at least in the NAc.

In the same genome-wide study that implicated E2F3 as a regulator of molecular responses to cocaine in the NAc, H3K4me3 enrichment was highly correlated with predicted E2F3 binding motifs, suggesting that this mark may be important in E2F3 action (Feng et al., 2014). We therefore tested whether overexpression of E2F3a in NAc increases H3K4me3 at this site in *Fosb* or if perhaps H3K4me3 is a pre-existing mark. No effect of E2F3a overexpression was observed on H3K4me3 enrichment (*F*_(1,40)_ = 0.057, *p* = 0.8126; *N* = 5–6; row l, Table 3). However, a main effect of gene position was observed (*F*_(3,40)_ = 37.61, *p* < 0.0001; row l, Table 3), with relatively high amounts of H3K4me3 enrichment near the E2F3 binding site 500 bp upstream of the TSS, as well as 250 bp upstream of TSS (Fig. 3*B*), indicating that H3K4me3 may be a permissive, pre-existing mark before E2F3 regulation of *Fosb*.

## Discussion

Here, we demonstrate that a specific isoform of the E2F3 transcription factor, E2F3a, is a novel regulator of the cocaine-elicited expression of the addiction-associated transcription factor ΔFosB. We show that in NAc specifically, E2F3 binding at the *Fosb* promoter at a site 500 bp upstream of the TSS is increased after repeated cocaine exposure. E2F3a overexpression in this brain region increases E2F3 binding at this same site and induces transcription of both *Fosb* products, *Fosb* and *ΔFosb* mRNA. Interestingly, we observed significant baseline binding of E2F3 500 bp upstream of TSS in PFC, VTA, and CPu but not regulation by cocaine. This result could point to a role for E2F3 in regulating steady-state *Fosb* expression in these reward circuitry regions, but one that is not affected by drug exposure. Further, using FACS, we determine that E2F3a’s regulation of ΔFosB is restricted to D1 MSNs in NAc, based on *E2f3a*’s restricted baseline and cocaine-induced expression in this cell type. There was also no effect of cocaine on much lower levels of *E2f3b* in either D1 or D2 MSNs. Based on overexpression experiments, we deduced that E2F3 binding in NAc, which cannot be differentiated by qChIP because of antibody limitations, is likely E2F3a, given that overexpression of E2F3b has no effect on expression of either *Fosb* isoform, whereas E2F3a increases expression of both isoforms. By contrast, miRNA knockdown of E2F3a is not sufficient to abolish the induction of ΔFosB following cocaine. Together, these results implicate E2F3a as a novel isoform- and region-specific regulator of ΔFosB, opening a new avenue for targeting this upstream regulator of addiction-mediated behaviors and a previously unrecognized signal transduction pathway affected in NAc by cocaine exposure.

Before this investigation, two studies implicated E2F3a expression in NAc as a mediator of the transcriptional, alternative splicing, and behavioral responses to cocaine (Feng et al., 2014; Cates et al., 2018b). The first, a genome-wide study by Feng et al. (2014), included a combination of ChIP-seq and RNA-seq and identified E2F3 as one of the most prominent upstream regulators of gene expression and alternative splicing in NAc after chronic cocaine exposure. The next, a study by Cates et al. (2018b), investigated specifically which E2F3 isoform is responsible for those changes. That study found that E2F3a, not E2F3b, acts in NAc as a novel regulator of gene expression, splicing changes, and behavioral responses induced by cocaine. Because it is known that ΔFosB, like E2F3a, potentiates cocaine reward (see Introduction), these recent findings with E2F3 suggested that it might be an upstream regulator of ΔFosB.

There are three critical points of regulation in the expression of ΔFosB: transcription of *Fosb*, alternative splicing of Exon IV, and the cell-type specificity of these events. Our findings add E2F3 to a list of several other factors that influence ΔFosB expression at different points in its production. PTB (encoded by *Ptbp1*) has been shown to regulate *Fosb* splicing *in vitro* (Alibhai et al., 2007). Specifically, increased PTB leads to preferential splicing of full-length *Fosb*, but when mutated causes decreased PTB binding at Exon IV and increased preferential splicing of *ΔFosb* (Marinescu et al., 2007). We did not find any direct involvement of E2F3a in the alternative splicing of *Fosb* and *ΔFosb*, as there was no evidence of binding at the Exon IV alternative splice site and no effect on the ratio of the two isoforms (Fig. 1*B*). Interestingly, E2F3a has been shown to regulate alternative splicing of *Ptbp1* itself (Cates et al., 2018b). Thus, it is possible that cocaine leads to alternative splicing of *Ptbp1* via E2F3a, increasing expression of a specific isoform of PTB that leads to the alternative splicing event giving rise to ΔFosB. However, prior work has shown no effect of chronic cocaine on *Fosb* splicing (Alibhai et al., 2007).

The transcription factors, CREB and SRF, have previously been shown to bind the *Fosb* promoter at −250 bp in response to cocaine exposure (Renthal and Nestler, 2008; Vialou et al., 2012). Deletion of both transcription factors together ablates the cocaine-mediated induction of ΔFosB, whereas deletion of either alone has no effect. Interestingly, deletion of both CREB and SRF reduces sensitivity to cocaine reward, whereas deletion of CREB alone enhances it, suggesting that each transcription factor differentially regulates ΔFosB expression in combination with other upstream regulators. These results are in agreement with the data presented in this study, in which viral-mediated knockdown of E2F3a is insufficient to abolish the induction of *ΔFosb* mRNA after an acute cocaine exposure (Fig. 2*F*).

E2F3a is expressed throughout the brain (Lein et al., 2007), and we observed significant binding of E2F3 at the −500 bp site on *Fosb* in NAc, PFC, VTA, and CPu. However, E2F3 binding to *Fosb* is specifically altered by cocaine exposure in NAc only (Fig. 1), indicating a role for E2F3 in the regulation of *Fosb* expression after cocaine exposure that is specific to NAc. Moreover, we show that expression of *E2f3a* is specific to D1 MSNs in NAc (Fig. 2*A*,*B*). Previous studies of ΔFosB have shown that it is expressed at baseline in both D1 and D2 MSNs, but that following both experimenter-administered and self-administered cocaine expression increases in D1 MSNs only (Lobo et al., 2013). These past findings align directly with our data presented here that indicate a D1 MSN-specific role for E2F3a in regulating the cocaine response in NAc.

In addition, another past study of ΔFosB’s function in D1 and D2 MSNs investigated the effects of overexpression in each cell type on cocaine-elicited reward. This investigation found that ΔFosB overexpression in D1 but not D2 MSNs in NAc was sufficient to cause cocaine conditioned place preference at a dose of 3.75 mg/kg (Grueter et al., 2013). Here, we find no detectable expression of *E2f3a* in D2 MSNs in NAc at baseline, and a D1 MSN-specific increase in the transcript elicited by chronic cocaine exposure (Fig. 2*A*,*B*). Together, these results suggest that E2F3a regulates ΔFosB expression in D1 MSNs following cocaine exposure, and thus may possibly play a role in ΔFosB-controlled changes in behavior and gene expression. Given that no *E2f3a* expression was detected at baseline in D2 MSNs, how ΔFosB is regulated in this cell type remains an outstanding question. Finally, our results support regional specificity for E2F3a’s function and suggest that it acts differently in distinct cell populations in different regions.

Given this MSN-specific and region-specific expression, our data support a broader understanding that ubiquitously expressed transcription factors target specific loci in different cells and specific brain regions. Despite this principle, it is interesting that we observed a non-significant decrease in E2F3 binding at the −500 bp site on *Fosb* in the CPu after cocaine exposure (Fig. 1*F*). NAc and CPu are differentially implicated in cocaine self-administration behaviors, with NAc being essential for the acquisition of lever-pressing and CPu being essential for the perseverance of behavior on more demanding schedules of reinforcement (Murray et al., 2012). ΔFosB is known to be induced in both regions after cocaine administration (Lobo et al., 2013). Given our findings, it would be worthwhile to investigate E2F3a’s regulation of ΔFosB expression in both NAc and CPu during different stages of rodent self-administration to determine how E2F3a and ΔFosB might play a role in the transition from volitional responding to compulsive drug-seeking.

That ΔFosB expression is specifically regulated in NAc by E2F3a, and not E2F3b, is also of note. The bioinformatic prediction of E2F3 as one of the most prominent upstream regulators of cocaine-induced gene expression and alternative splicing changes suggested a novel role for the E2F family in cocaine action but did not discriminate among family members or between these two E2F3 isoforms (Feng et al., 2014). E2F3a and E2F3b share the same DNA binding, nuclear localization, and transactivation domains, but E2F3b lacks an N-terminus ubiquitin-targeting domain (He et al., 2000). In proliferating tissue, E2F3a typically acts as a gene activator and E2F3b as a repressor (Leone et al., 2000). In fact, E2F3a was recently shown to be a novel regulator of cocaine elicited reward in that its overexpression enhanced cocaine reward and locomotor behaviors as well as recapitulated cocaine-induced gene expression and splicing changes (Cates et al., 2018b). Another recent study demonstrated a role for E2F3b in regulating cocaine action in the PFC (Cates et al., 2018a). Given these findings, our data on ΔFosB expression in post-mitotic brain tissue is consistent with divergent transcriptional regulatory roles for these two E2F3 isoforms.

In addition to predicting E2F3 as a novel upstream regulator, Feng et al. (2014) found significant enrichment of H3K4me3, a histone modification associated with active promoters in euchromatin, associated with E2F binding motifs in NAc in response to chronic cocaine administration. This finding suggests that, in response to chronic cocaine, H3K4me3 in NAc is either deposited as a chromatin mark that could permit E2F3 binding or that H3K4me3 is recruited by E2F3 in concert with its cocaine response to activate gene promoters. We tested these possibilities by overexpressing E2F3a in NAc and found that H3K4me3 enrichment at the *Fosb* promoter was unaffected. This result is consistent with the former hypothesis, that H3K4me3 is a pre-existing mark in NAc neuronal euchromatin that could allow E2F3a to bind its consensus sequence 500 bp upstream of the *Fosb* TSS and induce transcription. It is interesting to note that H3K4me3 is also enriched at baseline at −250 bp (Fig. 3*B*), the site where CREB and SRF bind their response elements to induce *Fosb* (Vialou et al., 2012); this implicates H3K4me3’s general involvement in *Fosb* transcription, though this speculation deserves further investigation in follow-up studies of *Fosb*’s transcriptional activators.

We did not investigate any other permissive or repressive chromatin marks that might interact with E2F3a at *Fosb* in NAc, such as H3K4me1, H3K27me3, or H3K27ac, among many others. Likewise, we did not investigate the status of H3K4me3 enrichment at *Fosb*’s −500 bp site in other brain reward regions. To this point, however, histone deposition data from the Roadmap Epigenomics Consortium shows enrichment of H3K4me3 at the −500 bp site upstream of the *Fosb* promoter in adult mouse forebrain, hindbrain, and midbrain (Zhou et al., 2011). This observation across brain regions is consistent with our observation that the mark is also enriched at this site under both baseline and chronic cocaine conditions in the NAc. This analysis indicates that the presence of H3K4me3 alone cannot explain why E2F3a can bind to and activate *Fosb* in NAc but not in the other brain regions studied. More targeted investigations are therefore needed to identify the histone modifications and perhaps other chromatin-remodeling factors that are responsible for E2F3a’s unique action on *Fosb* expression within D1 MSNs of the NAc.

In summary, we demonstrate a novel role for E2F3a, a specific isoform of the E2F family of transcription factors, in regulating the expression of ΔFosB downstream of cocaine action in NAc. ΔFosB is a critical regulator of the behavioral responses to diverse substances of abuse that can lead to addiction. Thus, understanding how ΔFosB is regulated by upstream transcription factors like E2F3a in the context of cocaine exposure will lead to a greater understanding of how this critical regulator might be exploited for potential therapeutic development in the treatment of addiction and other disorders related to the use of commonly abused substances.

## Synthesis

Reviewing Editor: Margaret McCarthy, Univ of Maryland School of Medicine Decisions are customarily a result of the Reviewing Editor and the peer reviewers coming together and discussing their recommendations until a consensus is reached. When revisions are invited, a fact-based synthesis statement explaining their decision and outlining what is needed to prepare a revision will be listed below. The following reviewer(s) agreed to reveal their identity: Lindsay De Biase, Weinan Sun.

This study advances the field by providing evidence supporting E2F3a as a novel upstream regulator for ΔFosB, an important molecular switch for addiction. These results extend the knowledge of the signaling network that involves ΔFosB in drug addiction and provide a potential therapeutic target for intervening addition and related disorders. With additional experiments, this manuscript would advance the field by showing a key role for the transcription factor E2F3 in regulating cocaine-induced changes in levels of fosB.

The authors use a variety of techniques to explore whether the transcription factor E2F3a plays a key role in regulating expression of FosB / ΔFosB in the nucleus accumbens following cocaine exposure. The manuscript is well-written and the data presented are sound, but don't go far enough to support the claim that they have demonstrated that this transcription factor is “a novel regulator of the cocaine-elicited expression of the addiction-associated transcription factor ΔFosB.” The manuscript presents several additional observations about how regionally specific this potential action of E2F3a is and whether there are links between E2F3a action and histone methylation status, but these observations feel partial and could be expanded to strengthen the manuscript.

Major concerns:

1.If the authors want to unequivocally demonstrate that E2F3a binding to the -500bp site on the Fosb gene plays a central role in cocaine-induced increases in FosB / ΔFosB, they need to do two critical things: 1) Show that repeated cocaine administration increases FosB / ΔFosB in the nucleus accumbens (although this has been shown repeatedly in the literature, they still need to show that this increase occurs in their own hands as a foundation for subsequent experiments) and 2) disrupt the E2F3a interaction with Fosb gene and show that this diminishes cocaine-induced increases in FosB / ΔFosB. Demonstrating that repeated cocaine exposure leads to increases in FosB / ΔFosB mRNA in nucleus accumbens can be accomplished via RT-PCR and the same primers the authors use for their assessment of these transcripts after viral overexpression of E2F3a and E2F3b. One feasible strategy for disrupting the E2F3a interaction with Fosb would be to use viral approaches to knock down E2F3a expression in the nucleus accumbens prior to / during cocaine administration and show that this diminishes cocaine-induced increases in FosB / ΔFosB (analogous to approaches used in Cates and Nestler 2017 to show that E2F3a played a key role in regulating cocaine-induced increases in FGFR1 for example). Additional experiments that could be carried out to support the assertion that E2F3a plays a key role in regulating cocaine-induced changes in FosB / ΔFosB could include:

a. Analyzing tissue after fewer cocaine injections. Presumably if E2F3a upregulation after cocaine (shown in Cates and Nestler 2017) and increased binding to the -500bp site on the Fosb gene strongly facilitates cocaine-induced changes in FosB / ΔFosB expression, E2F3a upregulation and increased binding should precede increases in FosB / ΔFosB during the time course of repeated cocaine administration. Demonstrating this would strengthen the claim of a potential causal relationship.

b. In general, is there a strong correlation between levels of E2F3 binding to the -500bp site and FosB / ΔFosB expression levels? The authors show that cocaine increases E2F3 binding to the -500bp site, but don't assess FosB / ΔFosB levels after cocaine exposure. Repeated cocaine exposure and viral E2F3a over expression appear to lead to similar magnitude of increased E2F3 binding to the -500bp site; it would be useful to be able to compare whether there are also similar levels of increased FosB / ΔFosB expression. i.e. do similar increases in magnitude of E2F3 binding lead to similar increases in levels of FosB / ΔFosB transcript?

c. Do increases in E2F3a transcript and increases in FosB / ΔFosB transcript occur in the same cell population after cocaine exposure? All of the analyses presented in the manuscript look at tissue level changes and both FosB and E2F3 are expressed by multiple neuronal populations as well as glial cells. If the same population of cells that show increases in E2F3a expression after cocaine (or viral overexpression) show increases in FosB / ΔFosB, this supports the idea that the two may be causally related. These sorts of experiments could be carried out with in situ hybridization (RNAscope) approaches.

2. The main conclusions of the manuscript critically depend on the qChIP data and their statistical analysis. In the figures, some data points lack error bars. For example, Fig. 1B IgG data points at -1250bp, Fig. 3A data point (red) at Exon IV, and Fig. 3B data points at -1250 bp all seem to lack error bars. In addition, please include the nature of repeats (n's meaning? how many animals?) in figure legends or in the statistical table for each data points shown in the figures. Since it is difficult to judge the data distribution from mean +/- s.e.m plots, consider showing individual data points along with the mean. Authors should also clearly define notations for statistical tests eg. ANOVA's F and t-test's t subscripts in the methods section.

3. in Figure 1B middle panel, the E2F3 saline group data points are consistently higher than the IgG negative controls. Please state whether these are significant.

4. in Line 181, authors state: “Interestingly, we observed a decrease in binding after cocaine in CPu (t8 = 1.45, p =0.185; Fig. 1E)”, as well as in Line 276, “Given this principle, it is especially interesting that we observed a trend toward a decrease in E2F3 binding at the -500 bp site on Fosb in the CPu after cocaine exposure (Fig. 1E).”

Since the p-value is 0.185, this difference is not significant. Indicating any trend is not statistically valid. Please add more repeats to this experiment in order to reject the null hypothesis. Alternatively, revise the manuscript's conclusions to reflect the saline and cocaine groups are not statistically different in CPu.

5. In figure 3B, the authors should also show the H3K4me3 enrichment without virus expression to exclude the possibility that this is HSV related.

6.The manuscript should include images to show that viral overexpression has been targeted to the proper brain region (low magnification images with the HSV-GFP to show effective targeting of injections to nucleus accumbens). The authors also need to demonstrate that over expression has been effective. This could consist of RT-PCR experiments to show that E2F3a and E2F3b transcript levels are elevated in the nucleus accumbens after viral injections and / or tissue level western blot to show that protein levels of E2F3a and E2F3b are elevated. Perhaps they did not see any effect of E2F3b on levels of FosB / ΔFosB transcript because the viral strategy to overexpress E2F3b didn't work for some reason. Without these data, the authors cannot use their current data as a basis to claim that E2F3a plays a key role in influencing FosB / ΔFosB while E2F3b does not.

Minor Suggestions:

1.The authors could bolster their findings by accessing the publicly available RNAseq data from Cates and Nestler 2017 to determine whether FosB / ΔFosB transcripts are also upregulated in their experiments involving E2F3a overexpression. Similarly, they could access publicly available ChIP-seq data for H3K4me3 from Feng and Shen 2014 and see whether H3K4me3 status at the Fosb gene (particularly around the -500bp site) is altered by chronic cocaine exposure. These data would represent additional, independent observations with distinct techniques that an important relationship may exist between methylation status at Fosb gene, E2F3a levels, and FosB / ΔFosB expression.

2.The authors are pitching E2F3a and potential downstream FosB / ΔFosB expression as unique therapeutic targets for changes induced during addiction. But E2F3a is expressed in many brain regions and in many cell types. Further, Cates and Nestler 2017 indicate that E2F3a overexpression alters expression levels of many genes, some, but not all of which show similar patterns of alteration to those induced by cocaine exposure. E2F3 is also implicated in changes associated with cell migration and cell cycle during development, with changes following genotoxic stress. Does this compromise our ability to think about this as a useful therapeutic target in the context of addiction? The authors should include more discussion of known functional roles of E2F3 in different contexts and what future experiments could solidify the idea that it might be a useful target in addiction. For example, making a mouse with alterations in the -500bp Fosb site that prevents E2F3 binding would allow experiments to show how disruption of this interaction (while allowing all other E2F3 interactions with other genes) impacts addiction related behaviors.

3.Is there anything different about the -500bp putative E2F3a binding site compared to the -250bp site or the ExonIV site that might explain why binding is more prominent here? Or are the putative binding sites literally identical in their sequence? The methods should probably include some brief description of how the MatInspector analysis works and it would be useful for the sequences of these putative binding sites and the non-binding -1250 site to be included in figure 1 so readers can see for themselves whether they are completely identical or nearly identical etc.

4.The abstract has too much methodological detail - we lose the overarching thrust of what is being proposed. In contrast, the results frequently jump immediately to statistics and don't include enough detail about the rationale for a particular set of experiments and how those experiments were carried out. For example, at the first point that cocaine administration is described in the body of the manuscript (first section in the results) the authors should describe exactly how cocaine was administered (dose, i.p. injection as route of administration, and number of days). Readers familiar with the addiction field will want to know this information right away without having to dig through the methods and readers not familiar with the field may fail to realize that repeated cocaine administration is being employed.

5.The authors need to be very cautious in how they word the significance statement and conclusions - identifying a potential upstream regulator of FosB / ΔFosB expression does not mean that you can automatically claim that upstream regulator is involved in drug-induced changes in behavior. It suggests that E2F3a may be upstream of behavioral changes but behavioral analysis would need to be carried out to after specifically disrupting the E2F3a - Fosb gene interaction in the context of drug exposure to fully support the idea that there is a functional relationship.

6.What is the status of H3K4me3 at the -500bp site of Fosb in PFC and VTA? If there are important regional differences, it seems like this might be a missed opportunity to explain why there aren't prominent cocaine-induced changes in E2F3 binding to the Fosb gene in these regions.
